# Kikuchi-Fujimoto Disease in an Iranian Woman; a Rare but Important Cause of Lymphadenopathy

**Published:** 2019-01-01

**Authors:** Mana Baziboroun, Masomeh Bayani, Ghodsieh Kamrani, Shahab Saeedi, Majid Sharbatdaran

**Affiliations:** 1 Infectious Diseases and Tropical Medicine Research Center, Health Research Institute, Babol University of Medical Sciences, Babol, Iran; 2 Department of Pathology, School of Medicine, Babol University of Medical Sciences, Babol, Iran

**Keywords:** Histiocytic necrotizing lymphadenitis, Fever, lymphadenopathy

## Abstract

Kikuchi-Fujimoto Disease (KFD), is a rare and self-limited condition of histiocytic necrotizing lymphadenitis, which typically presents as fever and lymphadenopathy. We describe a case of KFD in an Iranian woman. Due to low incidence and high importance, awareness of this disease is necessary for clinicians for early diagnosis and appropriate treatment. A 26-year-old woman was admitted to our hospital with a 3-week history of fever and lymphadenopathy. On physical examination, she had three separate enlarged lymph nodes on the right side of her neck. In laboratory tests that were carried out, she had mild anemia and an increase in C-reactive protein (CRP) level, erythrocyte sedimentation rate (ESR) and lactate dehydrogenase (LDH) level, while other tests were normal. Ultasound (U/S) guided core needle lymph node biopsy was performed and based on the histological finding, diagnosis of Kikuchi-Fujimoto disease was made. The patient was managed supportively and with prednisolone. She symptomatically improved and was discharged with no follow up. Although the incidence of KFD is rare, it must be considered as a differential diagnosis of lymphadenopathy especially in tuberculosis-endemic areas like our country-Iran. Moreover, it is necessary that physicians are aware of this disease in order to minimize unnecessary evaluation and toxic treatment.

## 1. Introduction

Kikuchi syndrome, also known as Kikuchi-Fujimoto disease (KFD) is histologically a histiocytic necrotizing lymphadenitis. The most common presentation of KFD is cervical lymphadenopathy with or without constitutional symptoms in previously healthy individuals. This disease often affects women under the age of 30, and is more common in Asia. It is self-limited and resolves spontaneously (1, 2). KFD is an extremely rare disorder. As a result, physicians' awareness of the disease is minimal (3). The exact cause of the Kikuchi disease is still unknown, but viral or autoimmune causes may have a role. However, the role of viruses is controversial (4).

KFD is of clinical significance, as it is confused with other illnesses like tuberculosis, lymphoma or systemic lupus erythematosus (SLE). No definite laboratory test for KFD has been found so far, as a result, diagnosis is to be confirmed through biopsy studies (5). Characteristic feature of Kikuchi disease in the excisional biopsy of involved lymph nodes is coagulative necrosis with plenty of karyorrhetic debris in paracortical areas (4).

In line with the above explanation, an immediate diagnosis and treatment of the disease is important since it prevents unnecessary further tests and patient's discomfort (6). In the following section, there is a case report of KFD in a young woman here in Babol, north of Iran.

## 2. Case presentation

A 26-year-old woman was admitted to our hospital with a three-week history of fever and painful lymphadenopathy. She initially had observed a painful swelling on the right side of her neck. Later, symptoms such as fever, nausea and fatigue were added. Despite the application of antibiotics such as amoxicillin/clavulanate and metronidazole, the swelling in the patient's neck had enlarged gradually. The patient didn't have any comorbidity. She was a married housewife, living with her family in a village around Babol-Mazandaran, Iran. In addition, she had no recent trip and no significant family history.

On the day of admission, she had stable hemodynamics, but further observation showed three separate enlarged lymph nodes on the right side of her neck, without any changes on the skin covering them. The woman was initially treated with clindamycin and naproxen. Anyhow, during two weeks of hospital stay, she had a fever ranging from 38-40 ◦C.

Laboratory studies demonstrated mild anemia (hemoglobin 10.9 gr/dL, normal range 14-18 gr/dL), with normal white blood cell count (6.4/ml, normal range 4.5-11/mL). Inflammatory markers were increased: C-reactive protein (CRP) level peaked at 77mg/l (normal range < 5 mg/l) and erythrocyte sedimentation rate (ESR) reached 42 mm/hour (normal range 0-20 mm/hour). The patient's serum lactate dehydrogenase (LDH) level had also increased to 519 U/L (normal range < 480 U/L). Cultures of blood and urine showed no growth and tuberculin skin test was negative. Serological tests detected evidence of previous Epstein-Barr virus (EBV) infection, but serologic tests for HIV, Hepatitis B and C, toxoplasmosis, venereal disease research laboratory (VDRL), ANA and RF were negative.

Chest radiography and computed tomography (CT) scan of the chest, abdomen and pelvis were unremarkable. Ultrasound (U/S) and CT scan of the neck, revealed multiple lymphadenopathy on both sides of the neck, expanding to the base of the neck and intra parotid. On the right side, lymph nodes in the posterior triangle of the neck, had pathologic morphology, with rim enhancement and hypodense center (central necrosis), up to 18 × 14 mm and on the left side, lymph nodes had a reactive view up to 12 × 5 mm (fig. 1).

**Figure2:**
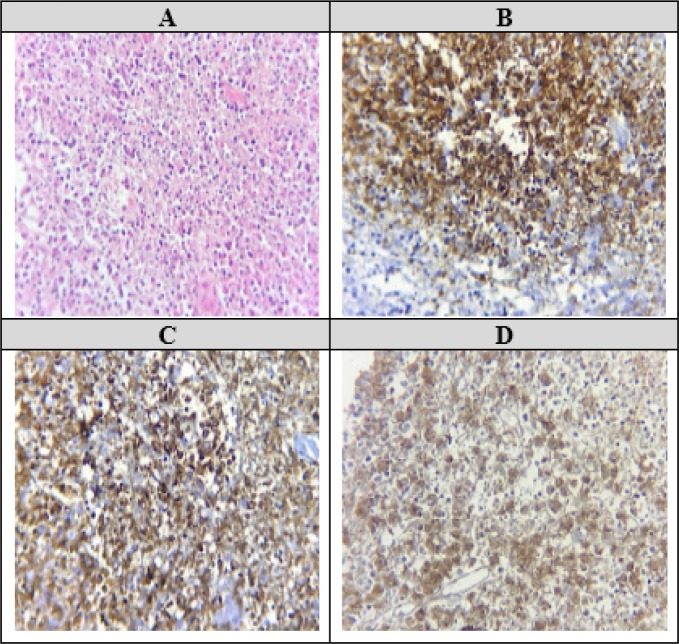
Histopathological findings of the lymph node. A) View of necrotic area and karyorrhectic debris (hematoxylin and eosin strain × 40). B) CD68+ histiocytes. C) CD3+ T-Cells. D) CD20+ B-cells

U/S guided core needle biopsy was performed, which revealed an effaced architecture with extensive coagulation necrosis and nuclear karyorrhexis, especially in the paracortical zone (fig. 2 A). No plasma cell or neutrophil was present. In an immunohistochemistry assessment, we found CD68-positive histiocytes in necrotic areas admixed with numerous CD3-positive T-cells (fig. 2 B&C). B-cells (CD20+) were also seen in remaining lymphoid follicles (fig. 2 D). These findings were compatible with Kikuchi's lymphadenitis.

The patient was managed supportively and with prednisolone. After 3 weeks, she symptomatically improved and was discharged with no follow up.

## 3. Discussion

Kikuchi-Fujimoto disease is a rare and benign disease that usually improves spontaneously. In a study performed on 244 cases of KFD, 70% of them were younger than 30 years old and 77% were women. Most of the patients come from East Asia (50%) (5, 7).

While the etiology of KFD remains unknown, infectious, genetic and autoimmune causes have been proposed. Various infections such as EBV, Herpes viruses, Hepatitis B, Parvovirus B19, Parainfluenza virus, HTLV1, Human immunodeficiency virus (HIV), Rubella, Paramyxovirus, Toxoplasmosis, Brucellosis, Bartonella henselae,and Yersiniaenterocolitica have been deemed related to the disease. Among them, EBV has been studied more, but its causal relationship has not been proved. Other reports have suggested autoimmune origin, especially SLE. Other autoimmune diseases associated with KFD are as follows: Still's disease, Sjogren's syndrome, Polymyositis and Rheumatoid arthritis. Studies have also found that interferon gamma, interleukin-6 and apoptotic cells probably play a role in KFD. Others have suggested certain food sources, like raw fish, as a cause (1, 8, 9).

The onset of Kikuchi disease can be acute or subacute and lasts about 2-3 weeks. Fever in 30-50% of cases is a primary symptom, but the main manifestation of the disease in approximately 80% of cases, is unilateral cervical lymphadenopathy, such as in our case (10). Although posterior cervical and supraclavicular nodes are involved most commonly, the involvement of other lymph nodes is noted uncommonly, such as axillary, inguinal and mediastinal nodes. Generalized lymphadenopathy is even more uncommon. Extra nodal extension of KFD has also been rarely reported, affecting skin, myocardium, bone marrow, C.N.S and eye movement. Systemic manifestations can also include malaise, headache, night sweats, weight loss and arthralgia (9, 11).

No specific laboratory test is available for Kikuchi syndrome. Anemia, leukopenia, raised ESR and CRP, and elevated lactate dehydrogenase and immunoglobulin levels can be found in this disease. Elevated levels of transaminases and ferritin are rarely found (11, 12). Our patient had mild anemia, elevated LDH and raised ESR and CRP, with normal transaminases.

Diagnosing the cause of the fever and lymphadenopathy, requires extensive work-up and considering differential diagnoses like lymphoma, tuberculosis, sarcoidosis, toxoplasmosis, cat-scratch disease, mononucleosis, AIDS and SLE; especially in countries where tuberculosis is endemic like Iran, KFD can be confused with tuberculosis clinically (1, 3, 13). Our patient had negative bacteriological cultures and negative viral serological tests. The result of tuberculin skin test was also negative. Clinical and laboratory characteristics in our patient were similar to lymphoma, but unlike lymphoma, enlarged lymph nodes were tender and pathologic results also ruled out lymphoma. The definite diagnosis of Kikuchi syndrome is made by excisional biopsy of affected lymph nodes and histological evaluation. Fine needle aspiration cytology (FNAC) is a cheaper, faster and less invasive method, but the accuracy is only 56%. Nevertheless, with clear clinical symptoms and the typical finding of KFD in FNAC, the diagnosis of Kikuchi disease can be done without lymphadenectomy (12), like what happened in our case. But if clinical finding is unusual, histopathological examination of lymph node by ultrasound-guided biopsy can be an optimal diagnostic method (3).

**Figuire1:**
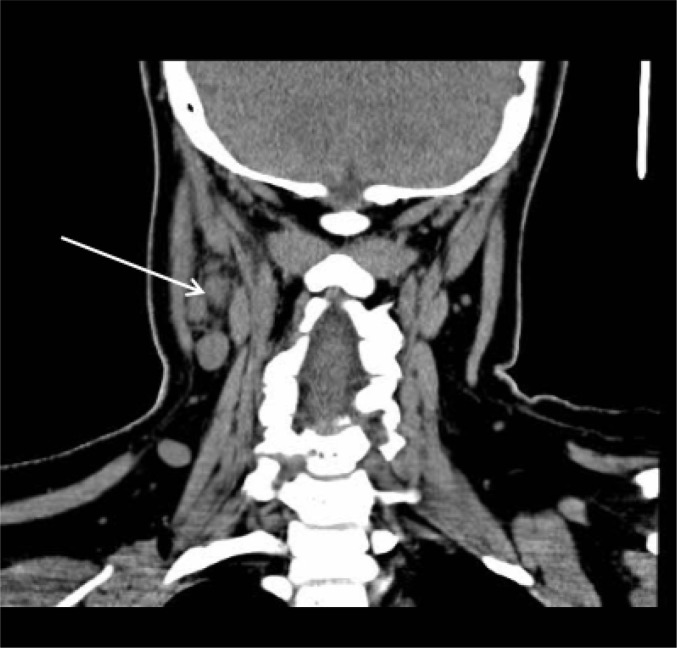
Computed tomography (CT) scan of the neck showing lymphadenopathy

Histological examination reveals paracortical foci of coagulation, necrosis and karyorrhetic debris. Histiocyte-associated antigens (lysosome, myeloperoxidase and CD68) are also expressed by histiocytes in KFD. However, for pathologists, differentiation of Kikuchi disease from other causes of lymphadenopathy such as SLE and lymphoma is challenging. The absence of plasmacells or neutrophils are other features in KFD, which rule out the diagnosis of SLE. Furthermore, absence of Reed-Sternberg or Hodgkin-like cells, is useful in ruling out leukemic process (10, 14).

Although Kikuchi disease is self-limited, has good prognosis, and usually resolves within 1-6 months, it can be recurrent (3-4% of cases) or become complicated with neurological manifestations, hemophagocytic syndromes or heart failure. Very rare cases of fatal outcome have also been reported (10). There is no specific treatment for KFD. The best approach is supportive and symptomatic therapy with analgesics and nonsteroidal anti-inflammatory drugs (NSAIDS). However glucocorticoids can be considered to reduce fever and painful lymphadenopathy, especially in generalized and complicated cases; furthermore, there are case reports of successful treatments with hydroxychloroquine, cyclosporine, azathioprine and intravenous immunoglobulin (1, 11).

## 4. Conclusions

This case report highlights the importance of Kikuchi disease as differential diagnosis of lymphadenopathy, especially in endemic areas for tuberculosis like our country; because this disease can be easily mistaken for TB, lymphoma and so on. So, although the incidence of KFD is rare, physician's awareness of this disease is the key to timely diagnosis and minimizing unnecessary evaluations and inappropriate treatment.
